# Development of Superior Fibre Quality Upland Cotton Cultivar Series ‘Ravnaq’ Using Marker-Assisted Selection

**DOI:** 10.3389/fpls.2022.906472

**Published:** 2022-05-24

**Authors:** Mukhtor M. Darmanov, Abdusalom K. Makamov, Mirzakamol S. Ayubov, Naim N. Khusenov, Zabardast T. Buriev, Shukhrat E. Shermatov, Ilkhom B. Salakhutdinov, Khurshida A. Ubaydullaeva, Jurabek K. Norbekov, Maftuna M. Kholmuradova, Sardor E. Narmatov, Ilyos S. Normamatov, Ibrokhim Y. Abdurakhmonov

**Affiliations:** Center of Genomics and Bioinformatics, Academy of Sciences of Uzbekistan, Tashkent, Uzbekistan

**Keywords:** QTL, SSR markers, traits, fibre, *Gossypium hirsutum*, marker-assisted selection

## Abstract

Marker-assisted selection (MAS) helps to shorten breeding time as well as reduce breeding resources and efforts. In our MAS program, we have targeted one of previously reported LD-blocks with its simple sequence repeat (SSR) marker(s), putatively associated with, at least, four different fibre quality QTLs such as fibre length, strength, micronaire and uniformity. In order to transfer targeted QTLs from a donor genotype to a cultivar of choice, we selected *G. hirsutum* donor genotypes L-141 and LN-1, possessing a fibre quality trait-associated LD-block from the chromosome 7/16. We crossed the donor lines with local elite *G. hirsutum* cultivars ‘Andijan-35’ and ‘Mekhnat’ as recipients. As a result, two segregating populations on LD-block of interest containing fibre QTLs were developed through backcrossing (BC) of F_1_ hybrids with their relative recipients (used as recurrent parents) up to five generations. In each BC and segregating BC_1_-_5_F_1_ populations, a transfer of targeted LD-block/QTLs was monitored using a highly polymorphic SSR marker, BNL1604 genotype. The homozygous cultivar genotypes with superior fibre quality and agronomic traits, bearing a targeted LD-block of interest, were individually selected from self-pollinated BC_5_F_1_ (BC_5_F_2–5_) population plants using the early-season PCR screening analysis of BNL1604 marker locus and the end-of-season fibre quality parameters. Only improved hybrids with superior fibre quality compared to original recipient parent were used for the next cycle of breeding. We successfully developed two novel MAS-derived cotton cultivars (named as ‘Ravnaq-1’ and ‘Ravnaq-2’) of BC_5_F_5_ generations. Both novel MAS cultivars possessed stronger and longer fibre as well as improved fibre uniformity and micronaire compared to the original recurrent parents, ‘Andijan-35’ and ‘Mekhnat’. Our efforts demonstrated a precise transfer of the same LD-block with, at least, four superior fibre QTLs in the two independent MAS breeding experiments exploiting different parental genotypes. Results exemplify the feasibility of MAS in cotton breeding.

## Introduction

The main goal of breeding programs is to mobilize genes from a donor genotype into an elite cultivar parent. Although traditional breeding methods showed efficiency in transferring a single gene/trait, it has limitations in the targeted-mobilization of multiple genes, regulating the complex quantitative traits (Abdurakhmonov et al., 2011). In particular, breeding of cotton cultivars with superior fibre quality is conventionally challenging because of multigenic regulation of quantitative trait loci (QTLs) for fibre quality as well as the existence of negative correlation among fibre quality traits, largely affected by a linkage drag (Lewis, 1962; Abdurakhmonov et al., 2008, 2009; Ijaz et al., 2019). In addition, the development of new cultivars using traditional selection methods is costly as well as time and resource-consuming task (Kushanov et al., 2021). To overcome these, DNA-based molecular markers are used in genetics and plant breeding to transfer traits of interest in a targeted manner, which is referred to as molecular breeding or marker-assisted selection (MAS; Abdurakhmonov, 2002; Collard and Mackill, 2008; Kushanov et al., 2021).

The molecular markers, based on the differences in the genetic material (DNA) sequence and closely located by a trait of interest, have evolved because of availability genetic sequence data. Molecular markers vary on types of genetic structure of DNA sequence and detection methods used to generate and record the polymorphisms between genotypes of interest (Collard et al., 2005; Amiteye., 2021). DNA markers associated with genomic regions of interest allow breeders to select plants early stage of plant growth based on a marker genotype rather than a phenotype that may get expressed later in plant vegetation. In particular, DNA markers are very useful and convenient for major QTLs, which are challenging task, if assessed by phenotypic evaluations during the course of breeding process. Therefore, DNA-based molecular markers and QTL-mapping results have become useful to find genome composition of interest (Tanksley et al., 1989). Usually, the targeted chromosomal segments of donor parents carry also undesirable traits along with a trait of interest. Because of power of trait-marker linkage association and linkage disequilibrium (LD) decay characteristics of neighbouring DNA markers over the genetic distance, DNA markers are the best instrumental for removing ‘undesired’ chunk of genetic material (linkage drag) coming from donor plant genome (Abdurakhmonov et al., 2008). In this case, DNA markers assist to minimize time to remove undesirable traits from F(n) generations after introducing QTL of interest into the recipient by a sexual crossing. Moreover, wild crops relatives have largely untapped source of desirable traits desirable alleles even with less expressed phenotypes (Migicovsky and Myles, 2017). Those can be identified by DNA markers and mobilized into elite germplasm to create new cultivars with superior phenotypes.

The huge number of QTL-mapping and genome-wide association studies in various cotton species and diverse sets of Upland cotton germplasms (He et al., 2007; Abdurakhmonov et al., 2008, 2009; Zeng et al., 2009; Zhang et al., 2012; Cai et al., 2014; Fang et al., 2014) have provided a portfolio of DNA markers, tightly linked with agronomically important cotton traits (Kushanov et al., 2021). Many QTLs for cotton fibre quality traits were identified for the last two decades through the mapping or association analyses of DNA markers in different populations or germplasm accessions in multiple environments (Sun et al., 2012; Liang et al., 2013; Fang et al., 2014; Shao et al., 2014; Zhiyuan et al., 2014; Said et al., 2015; Ma et al., 2019). However, a low level of functionality of many of these QTLs in different environments or breeding populations (Hugie et al., 2016) reduces their value in practical breeding (Fang et al., 2014). Loci that are detected in multiple environments are called stable and repeatable QTLs; therefore, they are favoured as reliable genetic loci for the MAS programs (Fang, 2018; Paudel et al., 2020). In this context, association studies, exploiting a large number of genotypes with simultaneous analyses of multiple alleles and majority of recombination events of genomic regions conditioning trait of interest, has become a powerful tool to mine stable QTLs for cotton breeding (Abdurakhmonov et al., 2008).

Sequencing efforts of several cotton genomes (diploid A and D, tetraploid AD; Pan et al., 2020) has provided a strong basis for accurate mapping of important QTLs useful for MAS programs. Moreover, genetic mapping in multiple populations using a large number of new generation molecular markers has led to reduce large gaps conventionally caused by the lack of polymorphism in certain complex genomic regions, helping to increase the number of mapped loci, confirm marker order, and increase marker coverage in cotton genome (Ulloa et al., 2017).

The competition for synthetic fibres, the variability of productivity from year to year, and the existence of new requirements for fibre quality due to technological developments in the textile industry continuously demand for novel cotton cultivars with superior fibre quality, fulfilling the industry requirements. The main fibre quality traits include fibre length (FL), fibre strength (FS), fibre uniformity (FU), fibre elongation (FE), and micronaire (FM) value. These are the most important characteristics that affect yarn quality (Yang et al., 2016). The application of MAS for the breeding of fibre quality traits along with yield potential is pivotal in cotton breeding (Li et al., 2016) although there is limited success of molecular breeding of fibre quality traits. To apply MAS tools for the improvement of fibre quality traits of commercially used Upland cotton of Uzbekistan, in this study, we exploited donor genotypes and fibre quality associated LD-block that was mapped using Simple Sequence Repeat (SSR) markers in our previous LD-based association study (Abdurakhmonov et al., 2009). As the result, two novel Upland cotton cultivars have been developed using MAS in a short period, with high fibre quality and agronomic potential. Our results should be helpful to accelerate cotton breeding programs, rapidly and precisely improving coarse fibred Upland cotton cultivars of Uzbekistan and timely responding the demand of national textile industry.

## Materials and Methods

### Plant Materials

Twenty-six donor germplasm accessions bearing LD-block/QTLs of interest were identified in associated mapping studies of Upland cotton germplasm in our previous studies (Abdurakhmonov et al., 2008, 2009). For our MAS program, we have chosen L-141 and L-N1 lines as unique donor parental lines, bearing QTLs for the four important fibre quality traits such as FL, FS, FU, and FM. The local commercial *G. hirsutum* cotton cultivars ‘Andijan-35’ and ‘Mekhnat’, genetically polymorphic to the donor, were used as recipient parents. These are local cultivars, widely grown by farmers in Uzbekistan, but have coarse fibre compared to the donor parents. In addition, the *G. hirsutum* elite cultivar ‘Namangan-77’, widely used as a breeding standard in Uzbekistan (Abdukarimov et al., 2003), were used as the control genotype for comparisons of fibre quality improvement. The ‘Namangan-77’ cultivar was also used as the negative control (BC hybrid - lack of QTL allele) for the course of MAS breeding process.

Sexual crosses were performed between ‘Andijan-35’ × L-141 and ‘Mekhnat’ × L-N1. Further, F_1_ hybrids were backcrossed up to BC_5_ generations, creating segregating populations on targeted QTL regions. In each BC and segregating BC_1_-_5_F_1_ populations, the transfer of targeted LD-block/QTLs was monitored using the early-season PCR screening of SSR marker (see below section) and the end-of-season fibre quality parameters. Only improved hybrids with superior fibre quality compared to original recipient parent were used for the next cycle of breeding. Subsequently, BC_5_F_1_ genotypes of interest were self-pollinated and BC_5_F_2_ generation plant populations have been developed. Homozygous cultivar genotypes with advanced for fibre quality, morphological and agronomical traits and bearing a targeted QTL/LD-block of interest were individually selected from BC_5_F_5_ population plants based on marker state and trait improvement as described above. More than hundred genotypes, carrying homozygous QTLs, were planted separately in ten meters’ rows as separate families. Breeding was performed based on homozygous state of SSR marker genotype, fibre quality and yield to obtain new ‘Ravnaq-1′ and ‘Ravnaq-2′ cotton cultivars.

### Plant Growth and Analysis of Fibre Quality Traits

The seeds of ‘Ravnaq-1’ and ‘Ravnaq-2’ were planted in one location with three replicates in a 90 × 15 × 1 plot scheme along with parental donor genotypes and control cotton cultivar ‘Namangan-77’ in three filed conditions with three replications during 2018 to 2020 years/seasons. Opened bolls from each replicate of all genotypes were individually harvested to analyse agronomic traits as harvesting materials. The traits such as 100 seed weight, and lint percentage were manually analysed in laboratory condition. The major fibre quality traits including FL, FS, FU and FM were measured using the High Volume Instrument (HVI; Uster Technologies, Inc., Knoxville, United States). Fibre quality traits of ‘Ravnaq-1’ and ‘Ravnaq-2’ cotton cultivars with their parent and control genotypes were studied over three years in three biological replications.

### SSR Marker Selection, Genomic DNA Isolation and PCR-Screening

**Figure 1:**
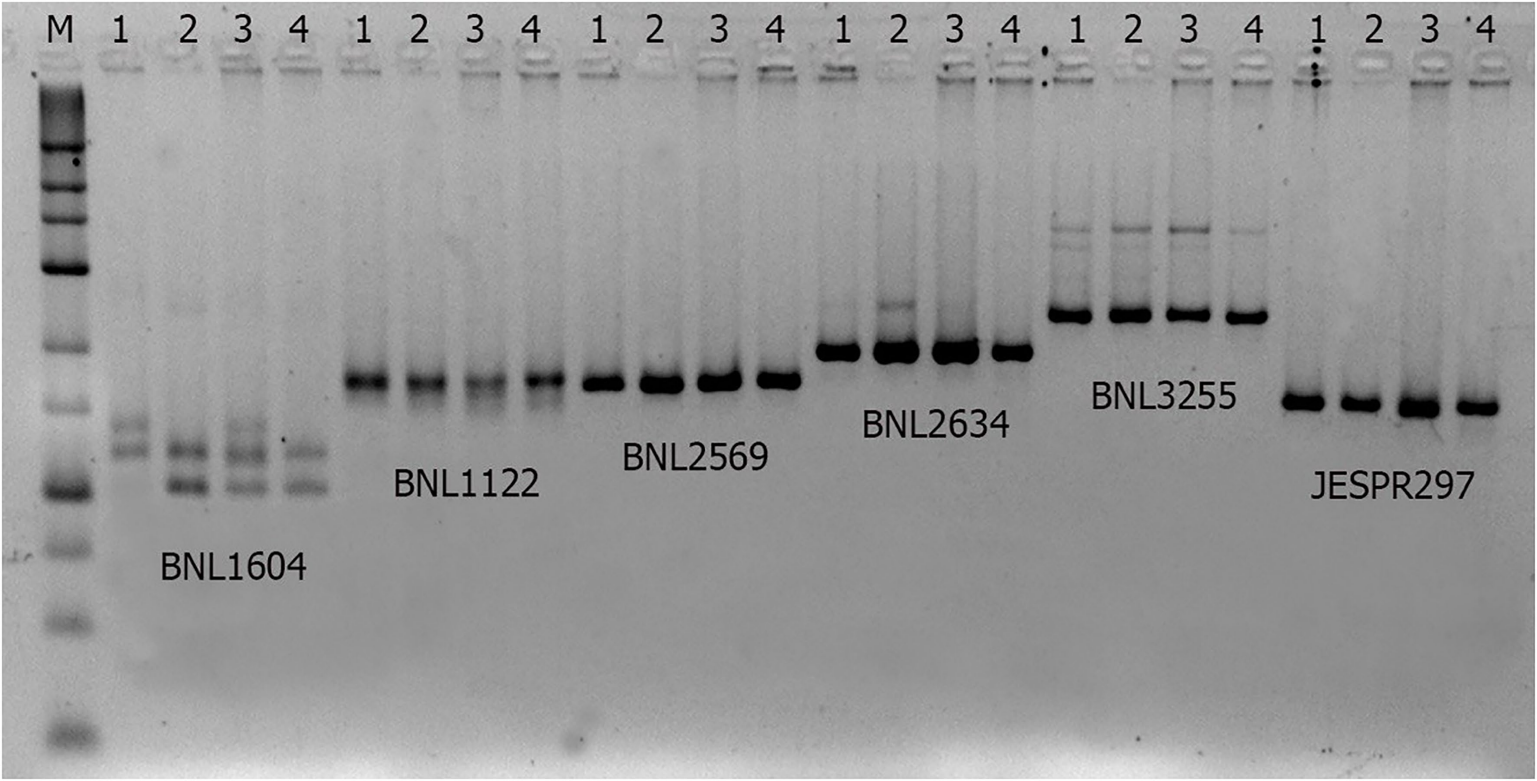
Agarose gel electropherogram of SSR markers in parental and hybrids. Parental genotypes (1 and 2), F_1_ hybrid (3) and BC generation hybrid (4).

We tested several SSR markers from our association study (Abdurakhmonov et al., 2009) in the donor and recipient parental genotypes (Figure 1; Table 1) and have chosen one of highly polymorphic SSR marker - BNL1604 (Figure 1) to use as a molecular tool to monitor the transfer of LD-block associated with fibre QTLs of our interest.

**Table 1:** Putative information on pairwise linkage, map distance, and QTL association of SSR markers in the targeted LD-block.

#	Marker-1	Marker-1_Chr_Loc: QTL^*^	Marker-2	Marker-2_Chr_Loc: QTL^*^	LD^*^, r^2^	Map distance between markers, cm	References
1	BNL1122_166	16: FL, FM	BNL1604_101	7: FL, FS, FM	0.06	–	Abdurakhmonov et al., 2009; Tan et al., 2015
2	BNL1122_170	16: FL, FU, FS, FM	BNL1604_101	16: -	0.05	4.6	Guo et al., 2007; Abdurakhmonov et al., 2009
3	BNL1122_174	7: -	BNL1604_101	7: FL, FS, FM	0.12	8.3	Yu et al., 2013; Tan et al., 2015
4	BNL1122_176	7: -	BNL1604_101	7: FL, FS, FM	0.17	8.3	Yu et al., 2013; Tan et al., 2015
5	BNL1395_156	16: FL, FM, LP	BNL1604_101	7: FL, FS, FM	0.05	–	Wang et al., 2011; Tan et al., 2015
6	BNL1395_160	16: FL, FM, LP	BNL1604_101	7: FL, FS, FM	0.05	–	Wang et al., 2011; Tan et al., 2015
7	BNL1604_134	7: FU	BNL1604_101	7: FL, FS, FM	0.38	–	Abdurakhmonov et al., 2009; Tan et al., 2015
8	BNL1604_136	7: -	BNL1604_101	7: FL, FS, FM	0.41	–	Tan et al., 2015
9	BNL1694_245	7: FL, FU, FS, FM, FE	BNL1604_101	7: FL, FS, FM	0.05	8	Tan et al., 2015
10	BNL1694_247	7: -	BNL1604_101	7: FL, FS, FM	0.04	8	Tan et al., 2015
11	BNL1694_247	7: -	BNL1122_166	16: FL, FM	0.08	–	Abdurakhmonov et al., 2009
12	BNL1694_247	7: -	BNL1122_170	16: FL, FS, FM, FU	0.08	–	Abdurakhmonov et al., 2009
13	BNL1694_237	7: -	BNL1122_170	16: FL, FS, FM, FU	0.06	–	Abdurakhmonov et al., 2009
14	BNL1395_162	16: -	BNL1122_172	16: -	0.76	0.7	Li et al., 2016
14	BNL1395_160	16: FL, FS, FM, FE	BNL1122_166	16: FL, FS, FM, FE	0.48	0.7	Shen et al., 2005; Abdurakhmonov et al., 2009; Li et al., 2016
16	BNL1395_158	16: -	BNL1122_168	16: -	1	0.7	Li et al., 2016
17	BNL1395_156	16: FL, RD	BNL1122_170	16: FL, FS, FM, FU	0.42	0.7	Shen et al., 2005; Abdurakhmonov et al., 2009; Li et al., 2016
18	BNL1395_160	16: FL, FS, FM, FE	BNL1395_156	16: FL, FM, RD	0.46	–	Abdurakhmonov et al., 2009
19	BNL1604_134	7: FU	BNL1122_174	7: -	0.11	8.3	Abdurakhmonov et al., 2009; Yu et al., 2013
20	BNL1604_134	7: FU	BNL1122_176	7: -	0.09	8.3	Abdurakhmonov et al., 2009; Yu et al., 2013
21	BNL1604_136	16: -	BNL1122_176	7: -	0.08	–	–
22	BNL1604_136	16: -	BNL1122_174	7: -	0.06	–	–
23	BNL1604_116	16: FL	BNL1122_166	16: FL, FM	0.11	6.3	Abdurakhmonov et al., 2009; Zhao et al., 2012
24	BNL1604_120	16: FL, FS, FM, FU	BNL1122_170	16: FL, FS, FM, FU	0.08	6.3	Abdurakhmonov et al., 2009; Zhao et al., 2012
25	BNL1604_116	16: FL	BNL1395_156	16: FL, RD	0.11	8	Wu et al., 2009; Abdurakhmonov et al., 2009
26	BNL1604_120	16: FL, FU	BNL1395_160	16: FL, FS, FM	0.09	11.9	Abdurakhmonov et al., 2009; Yu et al., 2013
27	BNL1604_132	16: -	BNL1694_245	7: FE	0.14	–	Abdurakhmonov et al., 2009
28	BNL1604_134	16: FU	BNL1694_245	7: FE	0.1	–	Abdurakhmonov et al., 2009
29	BNL1604_110	16: -	BNL1694_226	7: -	0.33	–	–
30	JESPR297_169	16: FL, FM, FU	BNL1395_166	16: FS	0.24	9.8	Wu et al., 2009; Mei et al., 2014; Tang et al., 2015
31	BNL1395_160	16: FL, FS, FM, FE	BNL1694_237	7: - FL, FU, RD	0.07	–	Abdurakhmonov et al., 2009; Wang et al., 2011
32	BNL1395_156	16: FL, RD	BNL1694_247	7: -	0.07	–	Abdurakhmonov et al., 2009
33	BNL1395_160	16: FL, FS, FM, FE	BNL1694_247	7: -	0.08	–	Abdurakhmonov et al., 2009

FL, fibre length; FS, fibre strength; FM, fibre micronaire; FU, fibre uniformity; FE, fibre elongation; RD, fibre reflectance.

* *r*^2^ ≥ 0.1 is a LD-decay threshold, all are significant at *p* ≤ 0.0001.

Leaf tissues were collected from all samples, and genomic DNAs were isolated from the frozen leaf tissues using the modified method of Dellaporta et al. (1983). Further, DNA concentrations were diluted in working solution (25 ng/μl) and stored in a refrigerator at −20°C. Amplification reactions were performed in 50 μl volumes containing 4.5 μl 10 × PCR buffer with 1.5 mM MgCl2, 1 μl BSA, 0.5 μl 25 mM of a dATP, dGTP, dTTP, and dCTP mix, 2.5 μl 50 ng/μl of microsatellite marker specific BNL1604 primers pairs (Supplementary Table 1) associated with fibre strength and fibre length, 1 μl of 25 ng/μl template DNA and 0.5 U Taq DNA polymerase (Sigma-Aldrich Chemie GmbH, Eschenstraße, Taufkirchen, Germany).

**Figure 2:**
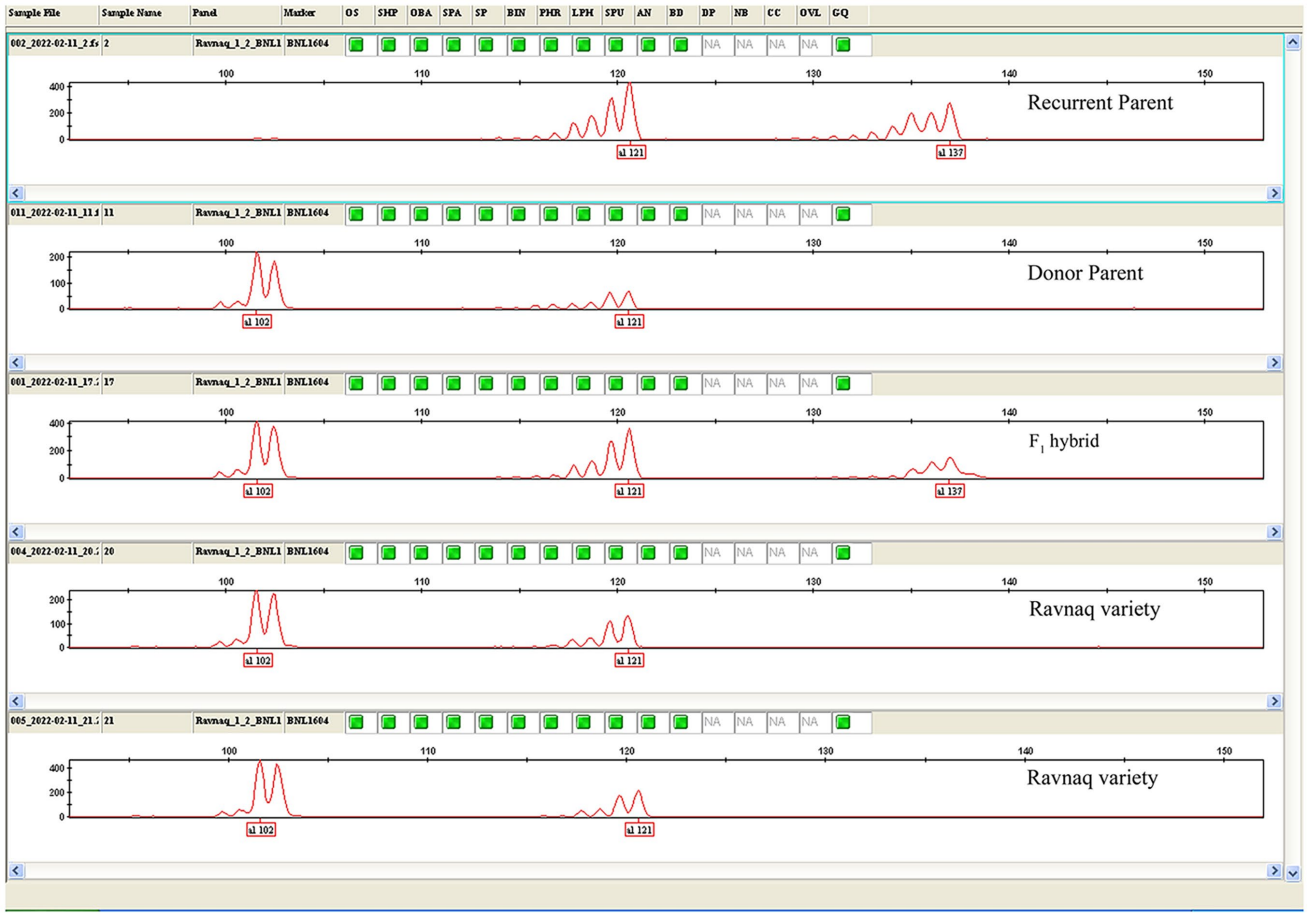
PCR amplicon separation for BNL1604 marker bands. ABI 3130XL Capillary electrophoresis results showing polymorphism between recurrent (with 121/137 amplicon) and donor [with 102/121 amplicon parents, F_1_ hybrid (102/121/137 amplicon)] and ‘Ravnaq’ cultivar (with 102/121 amplicon) for BNL1604–SSR marker PCR amplicons.

Amplifications were carried out with an initial denaturation at 94°C for 3 min followed by 45 cycles of 94°C for 20 s, 55°C for 30 s, and 72°C for 50 s. A final 5 min extension at 72°C was then performed. The procedures of PCR cycles were modified intentionally according to amplicon sizes (Abdurakhmonov et al., 2009). For determining PCR product sizes, 3.5% high-resolution agarose (Affymetrix, Inc., Cleveland, Ohio, United States) gel electrophoresis was carried out in 0.5 × TBE buffer. Gels were stained with ethidium bromide. In addition, the samples were run on ABI 3130XL Capillary electrophoresis (Life technologies, Carlsbad, CA, United States) to know the polymorphism between Recurrent and Donor parents, as well as F_1_ hybrid and ‘Ravnaq’ cultivars using fluorescent labelled BNL1604 (PET)—SSR marker (Figure 2). Microsatellite marker genotyping method (recipient genotype—a donor genotype—b and heterosis genotype—h) developed by Reddy et al. (2001) was implemented.

### Statistical Analysis

The variance and statistical analyses (Pearson’s Correlation, ANOVA, Two-Sample T-Test) were performed using the NCSS 2003 package software. The Kruskal–Wallis Multiple-Comparison Z-Value Test was performed to better identify the effects of the QTL allele introgressed by the MAS method. The HVI data obtained on fibre quality traits were analysed statistically according to the Pearson Correlation. The three-year data of fibre length and fibre strength characteristics obtained from each replication were analysed using the ANOVA and a comparative diagram was drawn in GraphPad Prism 8.01 (GraphPad Software, San Diego, CA, United States). Chi-square value (*χ*^2^) and broad sense heritability (H^2^) based on variations of FL and FS were analysed according to Petersen (1994) and Abdurakhmonov et al. (2005), respectively.

## Results and Discussion

As highlighted above, our previous LD-based association mapping study (Abdurakhmonov et al., 2009) have revealed several important SSR markers, which were in pairwise linkage disequilibrium state on the homoeologous chromosomes 7 (A_t_-genome) and 16 (D_t_-genome) of the allotetraploid cotton genome. These markers were putatively linked to the important QTLs (Table 1) in various experimental mapping populations. The genetic position of SSR markers associated with, at least, four important fibre quality QTLs (FL, ST, FM and FU) was 8–8.3 cM in chromosome 7 or 0.7–11.9 cM in chromosome 16, which were in a range of the estimated genome-wide LD-decay (LD-block) for cotton (Abdurakhmonov et al., 2008, 2009). This suggested an opportunity to mobilize this LD-block of interest through MAS using SSR marker of choice(s).

Most of fibre QTL-associated SSRs in this LD-block have amplified polymorphic SSR marker bands between donor lines bearing QTL and local cultivars or their hybrids, which were not easily distinguishable (Figure 1) on the commonly used agarose gel electrophoresis. Therefore, we have selected a highly visible polymorphic BNL1604 marker, among others, for our MAS program as the marker of choice to mobilize the targeted LD-block of our interest. Here, it is important to mention that BNL1604 marker showed statistically significant pairwise LD (r2 > 0.1; *p* < 0.0001) with other fibre quality associated SSR markers in the targeted LD-block of our interest (Table 1). In parental genotypes used for MAS, the BNL1604 SSR primer pairs have amplified three different polymorphic bands of 137 bp, 121 bp, and 101/102 bp. Its 101/102 bp marker band was highly distinguishable polymorphic amplicon between donor (L-141 and L-N1 lines) and recipient (‘Andijan-35’ and ‘Mekhnat’) genotypes (Figures 1, 2).

BNL1604, with 25-times repeated AG dinucleotide motive (AG25) and chosen as a marker of choice to carry MAS in this study, was developed by Brookhaven National Laboratory (BNL)^1^ in 2000. This SSR locus was widely used for genetic mapping studies (Table 1; Supplementary Table 2) to identify important QTLs in many different cotton species. Marker bands of this SSR was mapped in homoeologous chromosomes 7 and 16 and revealed a putative genetic linkage with FS, FL (Tan et al., 2015; Fang et al., 2021), FM, and FU. In addition, its association with agronomic traits such as lint percentage (LP), fruit branches and wilt resistance (Zhang et al., 2015) were reported in the literature (Supplementary Table 2). Interestingly, in most of the studies, BNL1604 primer pairs amplified two loci with 100/101 bp and 115/116 bp lengths. In our study (Abdurakhmonov et al., 2009), we observed seven marker bands of 101, 116, 120, 132, 134, 140, and 142 bp in *G. hirsutum* cultivar germplasm accessions. It is worth mentioning that several independent studies have reported somewhat unconcordant results on the size of amplicon bands and their chromosomal locations of BNL1604. For example, Guo et al. (2007) and Zhao et al. (2012) have mapped BNL1604_115 to the chromosome 7, while Wu et al. (2009) observed only BNL1604_116 locus and have located it to the chromosome 16. BNL1604_100 was located to the chromosome 16 (Guo et al., 2007; He et al., 2008; Zhao et al., 2012) while Gutiérrez et al. (2009) located BNL1604_101 to the chromosome 7 (Table 1). Our *in-silico* PCR analysis, using reference allotetraploid cotton genome, has clearly revealed that BNL1604_102 marker is on chromosome A07 with the DNA position of 89,031,336–89,031,437 (102 bp; data not shown).

**Figure 3:**
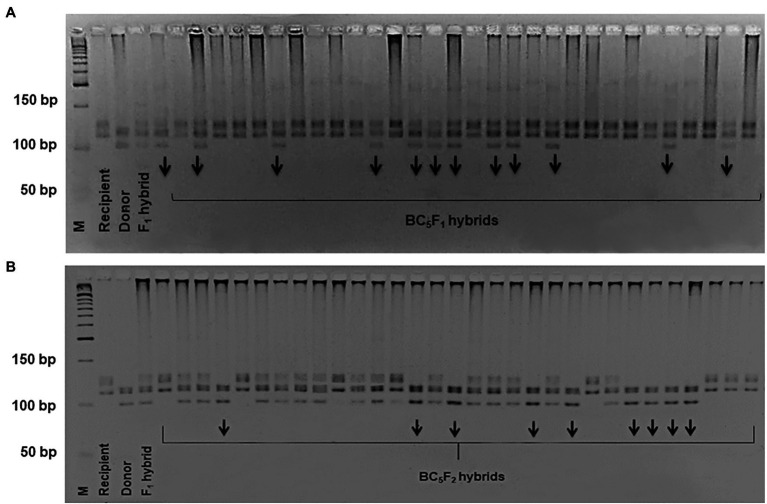
The electropherogram of BNL1604 marker in the BC_5_F_1_ **(A)** and BC_5_F_2_ **(B)** hybrid plants.

To mobilize the LD-block of our interest into the widely-grown Uzbek cotton cultivars, we crossed *G. hirsutum* donor genotypes L-141 and LN-1 possessing fibre quality QTLs on the chromosome 7/16, with local elite *G. hirsutum* cultivars ‘Andijan-35’ and ‘Mekhnat’ as recipients. F_1_ hybrids were backcrossed with their relative recipient genotypes (as recurrent parents) up to five generations to develop segregating populations on targeted QTLs. In each BC and segregating BC_1_-_5_F_1_ populations, segregation of BNL1604 genotype was within range of 1:1 ratio (*χ*^2^ ≥ 0.4, *p* ≥ 0.2; Table 2) of homozygous as recipient versus heterozygous as F_1_ hybrid genotypes. For example, in the BC_5_F_1_ [(‘Andijan-35’ × L-141) × ‘Andijan-35’] and BC_5_F_1_ [(‘Mekhnat’ × L-N1) × ‘Mekhnat’] hybrid populations with 188 and 164 plants, the heterozygous versus homozygous marker loci ratios was 96:92 and 78:86, respectively (Figure 3; Table 2). Only improved hybrids with superior fibre quality compared to original recipient parent were used for the next cycle of breeding based on end-of-season fibre quality measurements.

**Table 2:** The major fibre quality traits and genotyping of all MAS generations.

Plants	No.plant	FS	FL	Genotyping	Genotype ratio (a:h)	*χ* ^2^	*p*-value	a-genotype	h- genotype	b- genotype
**Andijan-35 × L-141**										
Recipient	30	32.2	1.13	A	–	–	–	–	–	–
Donor	30	36.8	1.27	B	–	–	–	–	–	
BC_1_F_1_	92	36.1	1.22	1:1	40:52	1.565	0.211	40	52	
BC_2_F_1_	92	35.8	1.20	1:1	41:51	1.087	0.297	41	51	
BC_3_F_1_	88	35.6	1.18	1:1	39:49	1.136	0.286	39	49	
BC_4_F_1_	141	35.9	1.20	1:1	63:78	1.596	0.207	63	78	
BC_5_F_1_	188	35.3	1.21	1:1	96:92	0.833	0.361	96	92	
BC_5_F_2_	235	36.2	1.18	1:2:1	58:116:61	0.115	0.944	58	116	61
**‘Mekhnat’ × L-N1**										
Recipient	30	29.4	1.12	A	–	–	–	–	–	–
Donor	30	38.7	1.21	B	–	–	–	–	–	–
BC_1_F_1_	89	35.4	1.2	1:1	49:40	0.910	0.340	49	40	–
BC_2_F_1_	54	35.2	1.18	1:1	25:29	0.296	0.586	25	29	–
BC_3_F_1_	60	35.8	1.19	1:1	28:32	0.267	0.606	28	32	–
BC_4_F_1_	134	34.9	1.19	1:1	74:60	1.463	0.227	74	60	–
BC_5_F_1_	164	35.3	1.21	1:1	78:86	0.833	0.361	78	86	–
BC_5_F_2_	191	36.2	1.18	1:2:1	44:99:48	0.424	0.809	44	99	48

Further, to get the homozygous cultivar genotypes, bearing the targeted LD-block/QTLs of interest, heterozygous BC_5_F_1_ plants in each combination were self-pollinated, resulting in BC_5_F_2_ segregating population for BNL1604 marker loci. In the BC_5_F_2_ [(‘Andijan-35’ × L-141) × ‘Andijan-35’] and BC_5_F_2_ [(‘Mekhnat’ × L-N1) × ‘Mekhnat’] combinations, 235 and 191 sample DNAs were screened by PCR. The results indicated the ration of 58:116:61 (1:2:1; *χ*^2^ = 0.115, *p* < 0.944) segregation in the combination of BC_5_F_2_ [(‘Andijan-35’ × L-141) × ‘Andijan-35’]. In that, 58 plants were homozygous (a) like the recipient parent, 116 samples were heterozygous (h) like the F_1_ hybrid and 61 samples were homozygous (b) like the donor parent. Similarly, in BC_5_F_2_ [(‘Mekhnat’ × LN1) × ‘Mekhnat’] combination, we observed monogenic marker segregation ratio of 44:99:48 (1:2:1; *χ*^2^ = 0.424, *p* < 0.809; Figure 3; Table 2). Genotyping analysis confirmed the stable monogenic Mendelian inheritance of the SSR marker band/LD-block in subsequent generations (1:2:1 segregation ratio with non-significant chi-square indices Table 2).

We also calculated the broad sense heritability (H^2^) based on variations of two major fibre quality trait data (FL and FS) of parental, BC_5_F_1_ and BC_5_F_2_ population genotypes of from above-mentioned two hybridization experiment (Table 3). Results revealed that in the cross of ‘Andijan-35’ and ‘Mekhnat’ as maternal parents and L-141 and L-N1 as paternal parents, 70 to 74% trait expression for FS was explained by the genetic variation, while 26–30%- trait variation was environmental. The broad sense heritability for FL was 0.53–0.54 in above combinations of parental lines and their hybrids, demonstrating 54% trait variation observed is explained by genetic loci transferred, while 46% trait variation is explained by environmental factors. Generally, a heritability value <20% is considered low, and a value >50% is considered high (Stanfield, 1983). As expected, most mapped QTLs corresponded to these characteristics with better genetic determination or stable heritability (Said et al., 2015). This showed suitability of the LD-block of our interest, exploited for fibre quality trait improvement using MAS.

**Table 3:** Parameters for the fibre strength (FS) and fibre length (FL) traits in parental. BC_5_F_1_ and BC_5_F_2_ crosses.

Plant type	Number of plants	Minimum	Median	Maximum	Mean	Std. Dev.	SE
Fibre strength (FS)							
Andijan-35	30	29.7	32.15	35.3	32.21	1.63	0.297
L-141	30	34.5	36.25	45.0	36.76	2.12	0.387
BC5F1(Andijan-35 × L-141) × Andijan-35	120	30.6	35.9	40.9	35.31	2.18	0.199
BC5F2(Andijan-35 × L-141) × Andijan-35	110	30.0	38.3	44.0	36.19	3.93	0.374
Fibre length (FL)							
Andijan-35	30	1.09	1.12	1.17	1.13	0.028	0.005
L-141	30	1.22	1.28	1.30	1.27	0.029	0.005
BC5F1(Andijan-35 × L-141) × Andijan-35	120	1.14	1.21	1.27	1.21	0.031	0.003
BC5F2(Andijan-35 × L-141) × Andijan-35	110	1.1	1.18	1.29	1.18	0.043	0.004
Fibre strength (FS)							
Mekhnat	35	28	28.7	35	29.35	2.13	0.59
L-N1	30	34.8	39.35	42	38.74	2.03	0.37
BC5F1(Mekhnat × L-N1) × Mekhnat	105	29.3	32.7	36.8	33.00	2.43	0.24
BC5F2(Mekhnat × L-N1) × Mekhnat	110	29.1	30.6	44.6	33.82	4.02	0.39
Fibre length (FL)							
Mekhnat	35	1.09	1.11	1.18	1.12	0.028	0.01
L-N1	30	1.17	1.21	1.27	1.21	0.029	0.01
BC5F1(Mekhnat × L-N1) × Mekhnat	105	1.13	1.18	1.26	1.19	0.028	0.00
BC5F2(Mekhnat × L-N1) × Mekhnat	110	1.1	1.14	1.28	1.17	0.042	0.00

**Figure 4:**
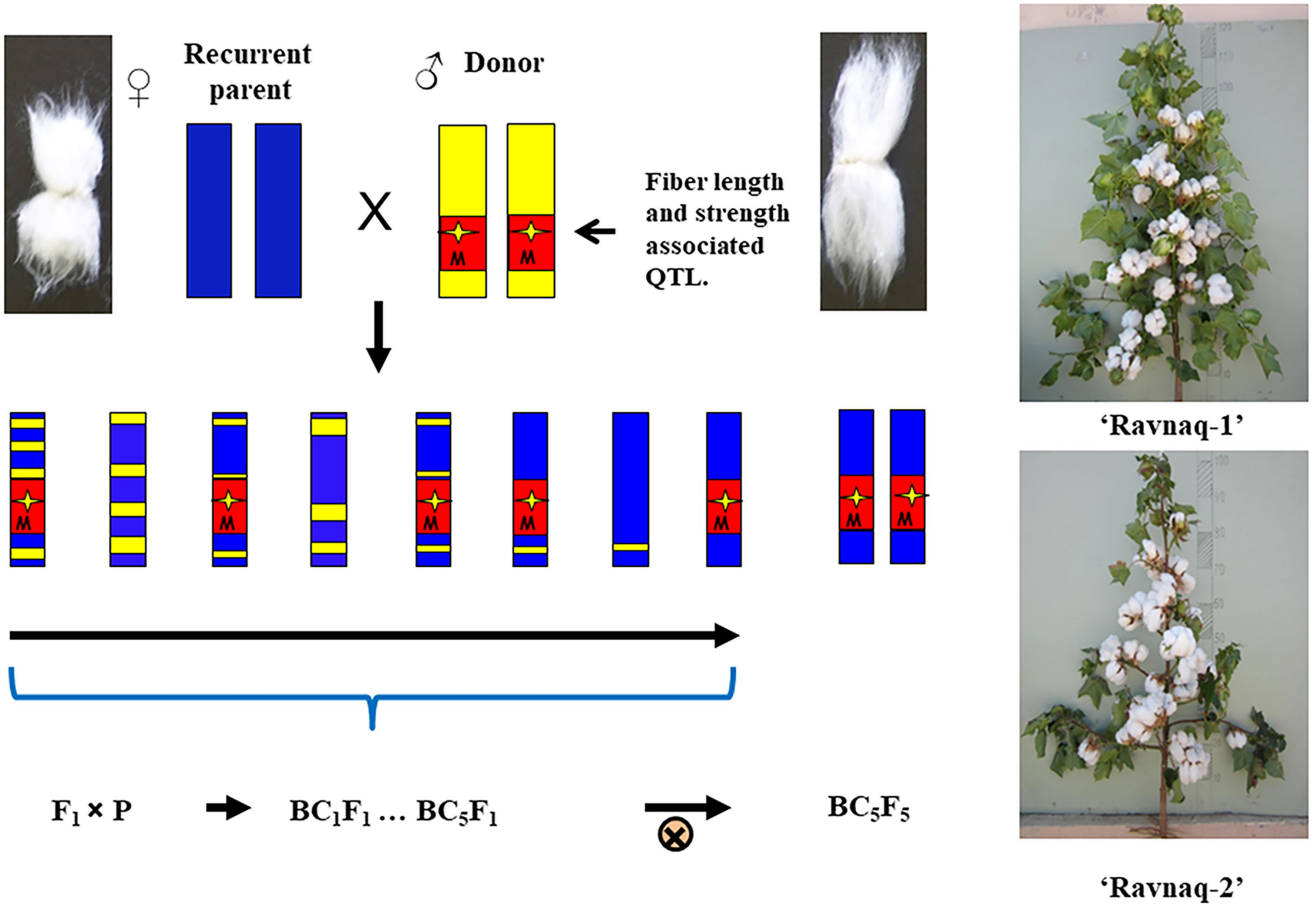
The scheme to obtain the ‘Ravnaq’ cultivar.

**Figure 5:**
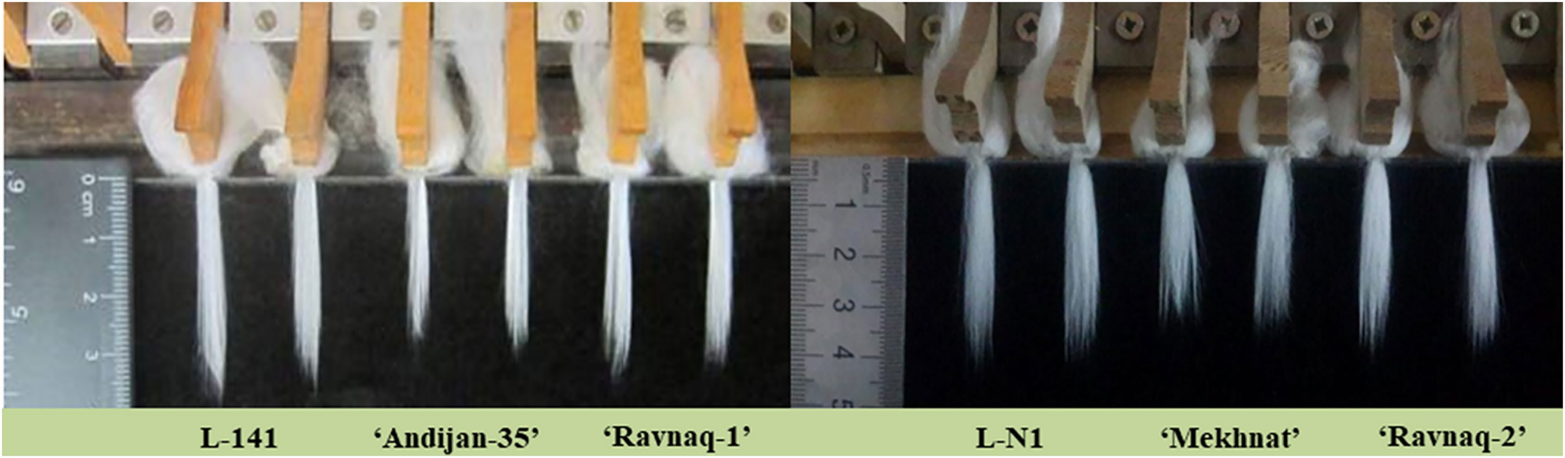
Fibre staple lengths (mm) of ‘Ravnaq-1’ and ‘Ravnaq-2’ cultivar compared to controls lines.

Further, only homozygous samples for introgressed QTL alleles from the donor genotype were selected from the BC_5_F_2_ segregating population of above-described two independent hybridization experiment. Homozygous genotypes from each crossing combinations were bred in separate breeding nurseries, focusing on fibre strength, length and uniformity, as well as other agronomic traits until the BC_5_F_5_ generations. As a result, based on the early-season PCR screening of SSR marker locus and the end-season fibre quality parameters in each generation, individual homozygous plant genotypes bearing homozygous SSR marker of donor parent were selected for the next season field studies and consequent seed increasing. From final plant families, two highly homozygous novel lines of BC_5_F_5_ [(‘Andijan-35’ × L-141) × ‘Andijan-35’] and BC_5_F_5_ [(‘Mekhnat’ × LN1) × ‘Mekhnat’] were selected as the stable MAS-derived cultivars. These novel genotypes have been named as ‘Ravnaq-1’ and ‘Ravnaq-2’, respectively (Figures 4, 5; ‘Ravnaq’ translates as ‘flourishing or advancing’). These new cotton cultivars were then submitted to the State variety testing commission of the Republic of Uzbekistan in order to conduct field trials in different soil-climatic conditions, which are currently underway.

**Figure 6:**
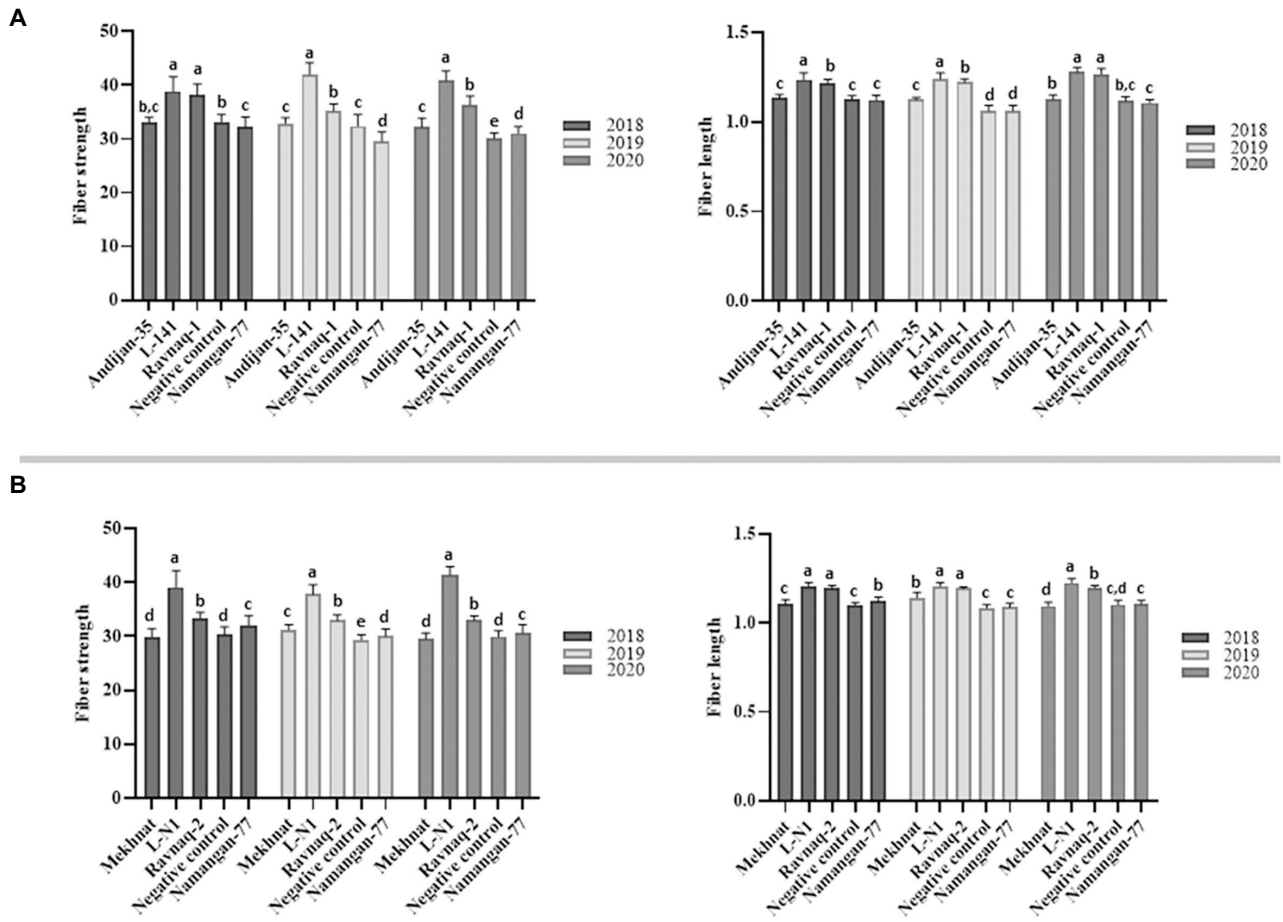
Comparative diagrams of fibre strength (FS) and length (FL) traits of ‘Ravnaq-1’ **(A)** and ‘Ravnaq-2’ **(B)** cultivars. The samples not connected by same letter are significantly different at *p* < 0.0001 [One-Way ANOVA, Tukey’s HSD (honestly significant difference) test].

Our result showed that ‘Ravnaq-1’, developed by crossing of high-yielded but low fibre quality commercial Upland cultivar ‘Andijan-35’ (recipient) with donor *G.hirsutun* L-141 line bearing the superior fibre quality QTLs, have acquired new fibre quality QTLs located within the targeted LD-block of interest, which were successfully selected using single BNL1604 marker. The comparative fibre quality analysis of all genotypes in the same environmental growing conditions showed distinct fibre quality characteristics. The ‘Ravnaq-1’ have shown the mean fibre length—1.23 inches and strength—36.8 g/tex. These parameters were significantly (*p* < 0.0001) lower in the same environment-grown original recipient genotype ‘Andijan-35’, in which the mean fibre length was 1.13 inches and strength—32.7 g/tex. The donor genotype L-141 cotton line had the mean fibre length and strength of 1.25 inches and 40.2 g/tex in the same growing condition, respectively. The negative control ‘Namangan-77’ had the mean fibre length of 1.11inches and fibre strength of 32.0 g/tex. These suggested that in ‘Ravnaq-1’ cultivar development, molecular marker of our choice has effectively selected superior fibre quality loci of the donor. The transferred LD block/QTL region has significantly (*p* < 0.0001) improved FL by 8.8% and FS by 12.5% compared to its recurrent parent ‘Andijan-35’ (Table 4; Figure 6). Moreover, we observed significant (*p* < 0.0001) improvement on FM and FU, demonstrating superior quality of MAS cultivar compared to original recipient parent.

**Table 4:** Fibre quality and yield traits of ‘Ravnaq-1’ **(A)** and ‘Ravnaq-2’ **(B)** averaged from 2018 and 2020 growing environments.

Traits^*^	L-141	Ravnaq-1	‘Andijan-35’	Namangan-77	Null hybrid
**(A)**					
**Fibre quality traits**					
FM (se)	4.16 (0.03)^d^	4.37 (0.019)^c^	4.76 (0.028)^a^	4.59 (0.026)^b^	4.70 (0.027)^a^
FS (se)	40.22 (0.237)^a^	36.79 (0.151) ^b^	32.70 (0.223)^c^	31.17 (0.205)^d^	31.96 (0.216)^d^
FU (se)	86.12 (0.128)^a^	85.88 (0.081)^a^	83.52 (0.12)^b^	83.6 2(0.111)^b^	83.41 (0.116)^b^
FE (se)	8.6 (0.067)^b^	9.4 (0.043)^a^	8.1 (0.063)^d^	8.4 (0.058)^c^	8.6 (0.061)^b^
FL (se)	1.25 (0.004)^a^	1.23 (0.002)^b^	1.12 (0.003)^c^	1.10 (0.003)^d^	1.10 (0.003)^d^
Staple Len (se)	37.69 (0.133)^a^	37.02 (0.085)^b^	33.27 (0.125)^c^	33.00 (0.115)^d^	33.37 (0.121)^c^
No. samples/replications	77/3	190/3	87/3	103/3	93/3
**Seed and lint percentage traits**					
Weight of 100 seeds. g (s.e.)	14.39 (0.02)^a^	13.59 (0.03)^b^	13.16 (0.03)^c^	11.94 (0.07)^e^	12.99 (0.03)^d^
Lint% (s.e.)	32.91 (0.29)^c^	35.56 (0.12)^d^	36.37 (0.11)^b^	37.13 (0.23)^a^	36.27 (0.23)^b^
					
Lint index (s.e.)	7.07 (0.09)^b^	7.50 (0.04)^a^	7.52 (0.04)^a^	7.06 (0.08)^b^	7.40 (0.07)^a^
No. samples/replications	30/3	30/3	30/3	30/3	30/3
**Traits**	**L-N1**	**Ravnaq-2**	**‘Mekhnat’**	**Namangan-77**	**Null hybrid**
**(B)**					
**Fibre quality traits**					
FM (se)	4.11 (0.029)^c^	4.39 (0.023)^b^	4.53 (0.027)^a^	4.58 (0.025)^a^	4.58 (0.026)^a^
FS (se)	39.66 (0.185)^a^	33.19 (0.149)^b^	30.15 (0.173)^d^	31.08 (0.164)^c^	30.00 (0.167)^d^
FU (se)	85.94 (0.142)^a^	85.04 (0.114)^b^	83.81 (0.132)^c^	83.64 (0.126)^c^	83.85 (0.128)^c^
FE (se)	8.49 (0.095)^b^	9.23 (0.076)^a^	8.25 (0.089)^c^	8.58 (0.084)^b^	8.47 (0.086)^b^
FL (se)	1.21 (0.003)^a^	1.20 (0.002)^b^	1.11 (0.002)^c^	1.11 (0.002)^c^	1.10 (0.002)^d^
Staple Len (se)	37.16 (0.116)^a^	36.45 (0.093)^b^	33.45 (0.108)^c^	32.97 (0.103)^d^	33.03 (0.104)^d^
No. samples/replications	81/3	126/3	93/3	103/3	100/3
**Seed and lint percentage traits**					
Weight of 100 seeds. g (s.e.)	14.13 (0.053)^a^	12.36 (0.067)^b^	12.40 (0.066)^b^	12.50 (0.06)^b^	11.94 (0.07)^c^
Lint% (s.e.)	32.40 (0.43)^c^	36.90 (0.17)^a^	37.38 (0.197)^a^	35.24 (0.15)^b^	37.13 (0.23)^a^
Lint index (s.e.)	6.94 (0.12)^c^	7.23 (0.046)^ab^	7.41 (0.092)^a^	6.84 (0.04)^c^	7.06 (0.08)^с^
No. samples/replications	30/3	30/3	30/3	30/3	30/3

* FE, elongation (or fibre elasticity, %); FM, micronaire; FS, fibre strength (g/tex); FL, fibre length or upper half mean (inches); FU, fibre uniformity (%).

Lint percentages ¼ (weight of lint fibres/weight of seed cotton) × 100; lint index ¼ (Lint percentage × weight of 100 seeds)/seed weight percentage. The samples not connected by same letter are significantly different at *p* < 0.0001 [One-Way ANOVA, Tukey’s HSD (honestly significant difference) test].

We observed similar results of fibre quality improvement in the other crossing combination experiment, where MAS-derived ‘Ravnaq-2’ successfully received novel fibre QTLs from the donor *G. hirsutum* line L-N1 bearing the LD-block of our interest and expressing a superior fibre quality compared to the recipient Upland cultivar ‘Mekhnat’. The results of fibre quality analysis of ‘Ravnaq-2’ under the same environmental conditions with its donor and recipient genotypes have shown the mean fibre length of 1.20 inches and strength of 33.2 g/tex. These parameters were significantly lower in the recipient genotype ‘Mekhnat’, in which the mean fibre length was 1.11 inches and strength—30.2 g/tex in the same growing condition. The donor genotype L-N1 cotton line had the mean fibre length and strength of 1.21 inches and 39.7 g/tex, respectively. The negative control had the mean fibre length—1.10 inches, fibre strength - 30.0 g/tex (Table 4). The trait improvement (*p* < 0.0001) in MAS-derived ‘Ravnaq-2’ genotype, by mobilizing the LD-block of interest using BNL1604 SSR marker, was 8.1% for fibre length and 9.9% for fibre strength, showing the genetic power of the novel QTLs (Table 4). The other two important fibre quality traits such as FM (4.39, *p* < 0.0001) and FU (85.04, *p* < 0.0001) have also improved compared to recipient parent ‘Mekhnat (4.53 and 83.81).

Further, in both MAS cultivars, we observed no significant changes or subtle but statistically significant improvements on seed and lint percentage traits, mainly acquired from the recipient parent genomes (Table 4). These suggested that MAS cultivars having better fibre quality have also kept some of other key trait parameters of the original recipient cultivars, attributing to the yield and other agronomic performance.

To establish the effect of genetically related QTL allele on fibre length and strength traits in ‘Ravnaq-1’ cultivar., parent samples, hybrids without marker allele (negative control), and standard ‘Namangan-77’ fibre quality parameters were analysed by Kruskal–Wallis Multiple-Comparison Z-Value Test method. According to the results of the analysis, the characteristics of fibre length and strength in ‘Ravnaq-1’ cultivar were significantly different (α = 0.05) from the recipient - ‘Andijan-35’ cultivar., Negative control, and standard cultivar ‘Namangan-77’ (Table 5). Similarly, the Kruskal–Wallis multidimensional comparison analysis results showed that the fibre length and strength parameters of the ‘Ravnaq-2’ cultivar were significantly higher (α = 0.05) than the parent lines and control cultivar genotypes grown in the same environments and/or bread with similar approaches (Table 6). These results have suggested the genetic role of mobilized LD-block/QTLs from the donors in trait improvements of MAS cultivars. Results also demonstrated the feasibility of MAS in the tetraploid cotton genome with a total recombinational length of about 5,200 cM using single or few markers (Abdurakhmonov et al., 2008), provided selection of polymorphic DNA marker from an LD-block of interest.

**Table 5:** Kruskal–Wallis Multiple-Comparison Z-Value Test method fibre length and strength in ‘Ravnaq-1’ cultivar.

FS^*^	‘Andijan-35’	L-141	Namangan-77	Null hybrid	Ravnaq-1
‘Andijan-35’	0				
L-141	11.8077	0			
‘Namangan-77’	2.9603	15.1246	0		
Negative control	1.343	13.2907	1.6131	0	
‘Ravnaq-1’	9.4878	4.5842	13.5604	11.2877	0
**FS**	**‘Andijan-35’**	**L-141**	**‘Namangan-77’**	**Null hybrid**	**‘Ravnaq-1’**
‘Andijan-35’	0				
L-141	11.068	0			
‘Namangan-77’	2.6431	14.0499	0		
Negative Control	2.0729	13.2463	0.5291	0	
‘Ravnaq-1’	10.7707	2.4982	14.5403	13.4603	0

* FS, fibre strength; FL, fibre length; ‘Andijan-35’, recipient genotype; L-141, donor genotype; ‘Ravnaq-1’ cotton cultivar; ‘Namangan-77’, standard cotton cultivar (control); Null hybrid, hybrids non-QTL allele.

Regular Test: Medians significantly different if *z* > 1.9600. Bonferroni Test: Medians significantly different if *z* > 2.8070.

**Table 6:** Kruskal–Wallis Multiple-Comparison Z-Value Test method fibre length and strength in ‘Ravnaq-2’ cultivar.

FS^*^	L-N1	‘Mekhnat’	‘Namangan-77’	Null hybrid	‘Ravnaq-2’
L-N1	0				
‘Mekhnat’	14.4536	0			
‘Namangan-77’	12.0712	2.8245	0		
Negative control	15.3471	0.6766	3.5722	0	
‘Ravnaq-2’	6.1330	9.6794	6.9201	10.6081	0
FL	**L-N1**	**‘Mekhnat’**	**‘Namangan-77’**	**Null hybrid**	**‘Ravnaq-2’**
L-N1	0				
‘Mekhnat’	11.7285	0			
‘Namangan-77’	12.3076	0.3164	0		
Negative control	13.9208	2.0716	1.8033	0	
‘Ravnaq-2’	1.8802	11.0801	11.7439	13.5385	0

* FS, fibre strength; FL, fibre length; ‘Mekhnat’, recipient genotype; L-N1, donor genotype; ‘Ravnaq-2’ cotton cultivar; Namangan-77, `standard cotton cultivar (control); Null hybrid, hybrids non-QTL allele.

Regular Test: Medians significantly different if *z* > 1.9600, Bonferroni Test: Medians significantly different if *z* > 2.8070.

Statistical analyses were performed using the Pearson method to study the genetic correlations among fibre quality parameters of MAS-derived genotypes of ‘Ravnaq-1’ and ‘Ravnaq-2’. In ‘Ravnaq-1’, a significant correlation (*p* < 0.001, *r* = 0.516) between the fibre strength and fibre length was obtained. Fibre strength (*p* < 0.001, *r* = 0.549) and fibre length (*p* < 0.001, *r* = 0.370) were also found to have a high and moderate positive correlation between fibre uniformity, and a weak positive or negative correlation between these two traits and other quality parameters were identified. Similar results were obtained by Wang X.Q. and others (2013) have shown a high positive correlation between fibre strength, fibre length (*p* < 0.01, *r* = 0.69^**^) and fibre uniformity (*p* < 0.01, *r* = 0.62^**^).

A low-value negative correlation was observed between FM and FL (*p* < 0.001, *r* = −0.248), FS (*p* < 0.001, *r* = −0.159), and FU (*p* < 0.001, *r* = −0.183). Genetic analysis of fibre quality parameters showed a significant high and moderate negative correlation between FM and FL (*p* < 0.001, −0.850) and FS (*p* < 0.029, −0.499) in the study of Yaqoob et al. (2016). According to the results of the Pearson correlation analysis, no opposite genetic correlation was observed between all fibre quality traits in the ‘Ravnaq-2’ cultivar at ≤0.05. For example, weak positive correlations were observed between FL and FS, FE and FU. In many other studies, a significant positive correlation between FL and FS and FE, as well as a negative correlation with FM were observed (Patil et al., 2014; Shang et al., 2016). A significant positive correlation was also observed between FS and FE, while a negative correlation was observed with FM. A similar positive correlation between FL and FS were observed in our previous exotic cotton germplasm studies (Abdurakhmonov et al., 2008). All these showed that fibre strength, elongation and uniformity become higher when fibre length is improved. Thus, it can be concluded that the correct correlation between the key fibre QTLs in the MAS-derived cultivars, described herein, is an additive effect of the QTLs and/or a joint effect of genes transferred from the donor genotypes to the cultivars. The positive correlation between lint percentage and FL in our MAS-derived cultivars can also be explained by the fact that the correct breeding approach was carried out in each backcrossed hybrid on FL and lint percentage parameters.

The ‘Ravnaq-1’ and ‘Ravnaq-2’ cotton cultivars were first planted on 26 and 30 hectares of special seed increase farms in the Namangan region. In the cultivation of these cultivars, all agrotechnology measures were taken in a timely manner, which allowed farmers to get the seed cotton yield of 4.1 tons per hectare. In order to conduct field trails on larger areas in 2018, the ‘Ravnaq-1’ cultivar was planted on 500 hectares in the Namangan region, which allowed to obtain an average of more than 4.0 tons per hectare of seed cotton yield in the region. In 2019, the ‘Ravnaq-1’ cotton cultivar was planted in large areas in the Tashkent region, with an average yield of more than 3.6 tons per hectare of seed cotton yield in the region. Moreover, ‘Ravnaq-1’ has been planted in Surkhandaryo and Syrdaryo regions since 2021, and ‘Ravnaq-2’ cotton has been planted on farms and agriculture clusters in the Republic of Karakalpakstan, which is a relatively northern region of Uzbekistan. We observed high yield (3.8 tons per hectare of seed cotton yield) with high fibre quality in Karakalpakstan environment (data not shown). The seed cotton yield of MAS cultivars grown in the farmers field condition was competitive to those widely commercialized Upland cotton cultivars, including the recipient cultivars (average seed cotton yield of Uzbekistan cultivars is in range of 2.6 to 3.6 tons per hectare; data not shown). Some important characteristics of ‘Ravnaq’ cultivars are sown in Supplementary Figures 1, 2. These results demonstrated that novel MAS cultivars, in addition to newly acquired superior fibre quality traits, have competitive agronomic properties for sustainable farming in Uzbekistan.

## Conclusion

Our research on the use of the first DNA-based MAS in Uzbekistan to improve one or more fibre quality traits in Upland cotton cultivars has proven to be a successful practice that led to the improvement of important fibre quality traits in widely-grown local Upland cultivars. Molecular marker BNL1604, chosen from the specific LD block associated with fibre QTLs along with donor genotypes, identified in our previous associative mapping studies using Upland cotton germplasm, were practically useful for our MAS program. We showed here that the mobilization of novel QTLs using SSR markers is effective to improve the key fibre quality traits such as FL, FS, FU, and FM. New cultivars, ‘Ravnaq-1/Ravnaq-2’, registered in the State Variety Testing Commission of Uzbekistan in 2014–2017 are the first generation of MAS-derived cotton cultivars in Uzbekistan. Our results highlight that LD-block of chromosome 7 with its mapped molecular marker(s) and donor genotypes, used herein, have efficiently helped to precisely and rapidly transfer superior fibre quality QTLs to the commercially grown Upland cotton cultivars, exemplifying the potential of MAS in cotton breeding.

## Data Availability Statement

The original contributions presented in the study are included in the article/Supplementary Material, further inquiries can be directed to the corresponding authors.

## Author Contributions

IA, MD, AM, MA, and ZB conceived and designed the study. MD, AM, KU, JN, MK, SN, and IN carried out field experiments. MD, AM, NK, SS, ZB, and IS performed data analyses. MA, AM, and IA drafted the manuscript. All authors contributed to manuscript revisions and approved the submitted version.

## Funding

This research was funded by the Ministry of Innovative Development of Uzbekistan providing the research project (A6-T029, FA-F5-T030, IFA-2017-5-6, A-FA-2021-474).

## Conflict of Interest

The authors declare that the research was conducted in the absence of any commercial or financial relationships that could be construed as a potential conflict of interest.

## Publisher’s Note

All claims expressed in this article are solely those of the authors and do not necessarily represent those of their affiliated organizations, or those of the publisher, the editors and the reviewers. Any product that may be evaluated in this article, or claim that may be made by its manufacturer, is not guaranteed or endorsed by the publisher.

## Footnotes

^1^ www.cottongen.org

## Supplementary Material

Supplementary Figure 1 Some morphological phenotypes of MAS-derived ‘Ravnaq-1’ cultivar.

Supplementary Figure 2 Some morphological phenotypes of MAS-derived ‘Ravnaq-2’ cultivar.

## Abbreviations

QTL: Quantitative trait loci
MAS: Marker-assisted selection
SSR: Simple sequence repeats
NIL: Near isogenic lines
BC: Back crossing
FS: Fibre strength
FL: Fibre length
FE: Fibre elongation
FM: Fibre micronaire
FU: Fibre uniformity
‘Ravnaq’: Translates in English as flourishing or advancing
