# Supplementary material for: Development of Superior Fibre Quality Upland Cotton Cultivar Series ‘Ravnaq’ Using Marker-Assisted Selection
Source: Front Plant Sci. 2022 May 24;13:906472. doi: 10.3389/fpls.2022.906472 (PMC9168987; doi:10.3389/fpls.2022.906472)
Supplement: Supplementary file 1 [file Table_1.docx]

**Supplementary Table 1. Primer pairs used for PCR-amplification.**

| Primer name | Orientation | Sequence (5'-3') | Repeat Motif | Length (nt) |
| --- | --- | --- | --- | --- |
| BNL1604 | Forward | AGAGGGAGTAAAGATTTGGGG | (AG)25 | 21 |
|  | Reverse | TCCAGTTCTTTTTGCCTTGG |  | 20 |
| BNL1122 | Forward | TCGATAACGGCTATAGTAATCTCTC | (AG)16 | 25 |
|  | Reverse | CAACAAATAAGCAGCCAAGAAA |  | 22 |
| BNL2569 | Forward | CAGAGAGCCATTGTGAACGA | (GA)13 | 20 |
|  | Reverse | ATAATGCTAGGGCATGTGGC |  | 20 |
| BNL2634 | Forward | AACAACATTGAAAGTCGGGG | (AG)11 | 20 |
|  | Reverse | CCCAGCTGCTTATTGGTTTC |  | 20 |
| BNL3255 | Forward | GACAGTCAAACAGAACAGATATGC | (GC)6AT(AC)14 | 24 |
|  | Reverse | TTACACGACTTGTTCCCACG |  | 20 |
| JESPR297 | Forward | GAGAACTCGTTAAAGCACAATG | (GAA)12 | 22 |
|  | Reverse | GTTAATAGAGTTGGGTTTCTCATG |  | 24 |
