# Supplementary material for: Development of Superior Fibre Quality Upland Cotton Cultivar Series ‘Ravnaq’ Using Marker-Assisted Selection
Source: Front Plant Sci. 2022 May 24;13:906472. doi: 10.3389/fpls.2022.906472 (PMC9168987; doi:10.3389/fpls.2022.906472)
Supplement: Supplementary file 2 [file Table_2.docx]

**Supplementary Table 2. The quantitative and agronomic traits association studies of BNL1604 microsatellite marker.**

| **Traits** | | **QTL name** | **Marker interval** | **Chr.#** | **Material type** | **References** |
| --- | --- | --- | --- | --- | --- | --- |
| Fiber quality | FL | - | FL QTL close to BNL1604 | 7 | - | He et al., 2007 |
|  | FL, FU | - | - | - | Uzbek germplasm collection | Abdurakhmonov et al., 2009 |
|  | FL, FS, FM | qFL07-1, qFS07-1, qFM07-1 | - | 7 | - | Tan et al., 2015. |
|  | FE, FM, FS, FU | qFE07-1, qFM07-1, qFS07-1, qFU07-1 | BNL1604 located close to QTLs for FE, FM, FS, and FU | 7 | - | Zhang et al., 2012. |
|  | FL, FM, FS, FU | qFM-c7-1, qFS-c7-1, qFU-c7-1 | TMB1618-BNL1604, BNL1604-NAU1085, TMB1618-BNL1604 | 7 | F2, F2:3 and RIL population derived from crosses between sGK9708X0-153. | Sun et al., 2012. |
| Agronomic data | LP % | qLP-08A-c-7-1 | BNL1694-BNL1604 | 7 |  | Yu et al., 2013. |
|  | LP % | - | BNL1604_98 bp | - | Exotic population derived through species polycrosses | Zeng et al., 2009. |
|  | FL, FM and LP% | qFL-16-1, qFM-16-1, qLP-16-1 | BNL1395–BNL1604; BNL1604; BNL1395–BNL1604; BNL1604 | 16 | F2 and F2:3; F8; F2:3; F8; F2:3 | Wang et al., 2011. |
|  | Effective fruit branches - EFB | - | - | 16 | Cotton cultivars | Li et al., 2016 |
|  |  |  |  |  |  |  |
|  | Wilt Resistance: VW, FW | - | - | 16 | BIL population | *Zhang et al., 2015. |
|  | FS, FL, FM, FU | qFS07.1_FL, FS, FM, FU. qFS07.1 and qFS14.1 | FM07.1 and qFM08.1 | 7 | RIL population | Tan et al., 2015. |

*
