# Supplementary figures and images for: Development of Superior Fibre Quality Upland Cotton Cultivar Series ‘Ravnaq’ Using Marker-Assisted Selection
Source: Front Plant Sci. 2022 May 24;13:906472. doi: 10.3389/fpls.2022.906472 (PMC9168987; doi:10.3389/fpls.2022.906472)

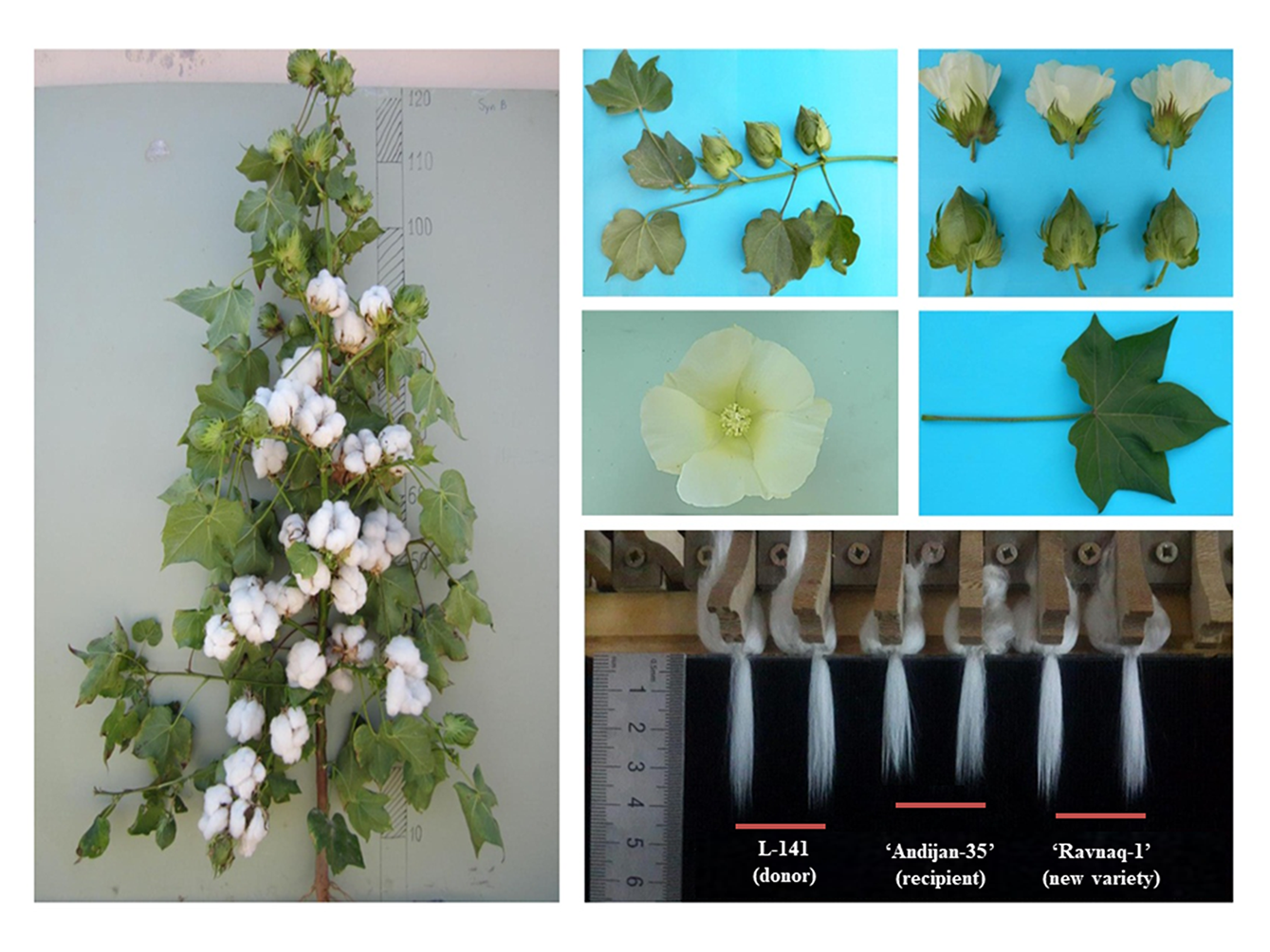

Supplement: Supplementary Figure 1 — Some morphological phenotypes of MAS-derived ‘Ravnaq-1’ cultivar. [file Image_1.TIF]

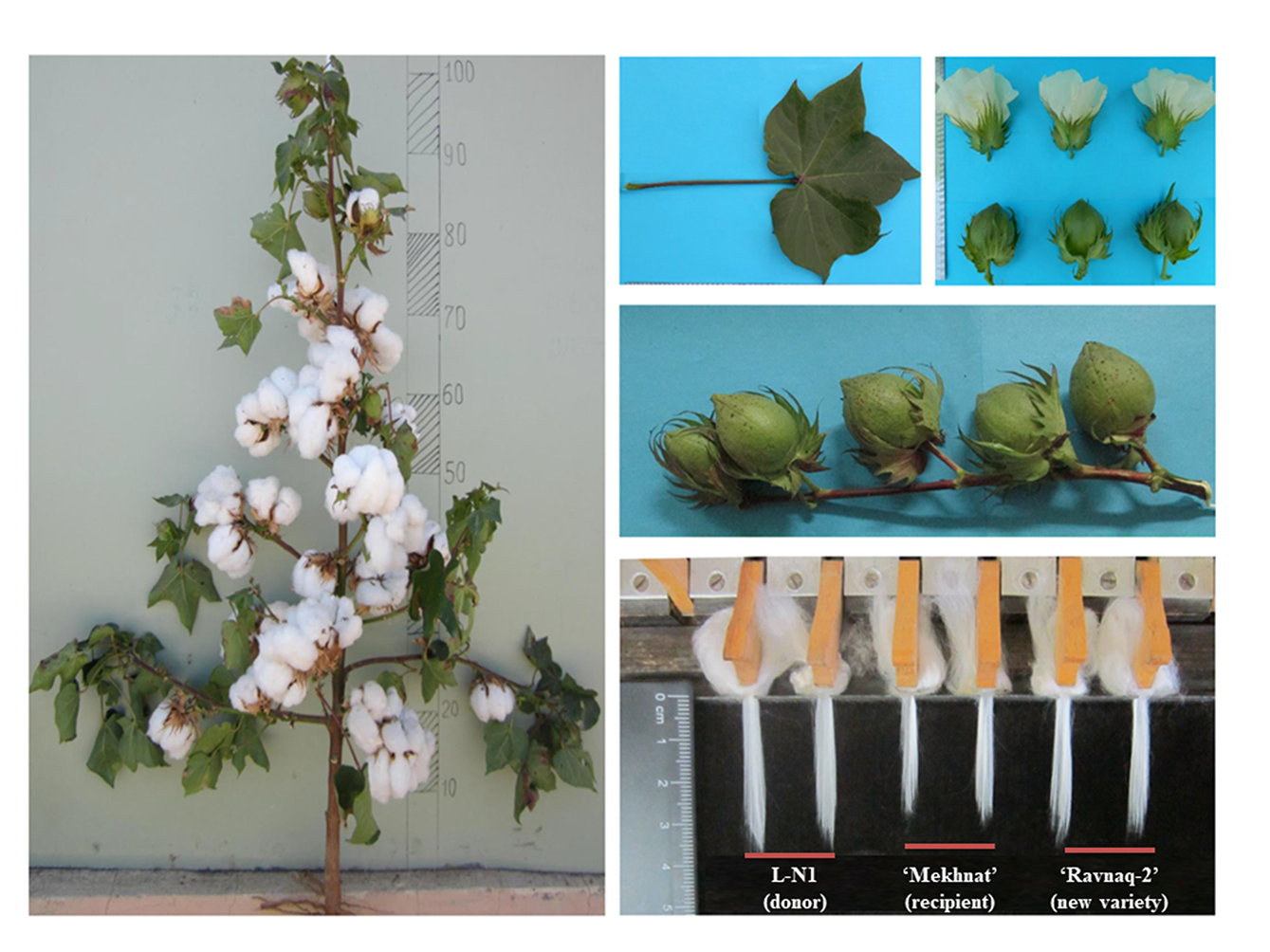

Supplement: Supplementary Figure 2 — Some morphological phenotypes of MAS-derived ‘Ravnaq-2’ cultivar. [file Image_2.TIF]
